# Plasma triglycerides predict ten-years all-cause mortality in outpatients with type 2 diabetes mellitus: a longitudinal observational study

**DOI:** 10.1186/s12933-014-0135-6

**Published:** 2014-10-11

**Authors:** Maria-Agata Miselli, Edoardo Dalla Nora, Angelina Passaro, Franco Tomasi, Giovanni Zuliani

**Affiliations:** Department of Medical Sciences, Section of Internal and Cardiorespiratory Medicine, University of Ferrara, Ferrara, Italy; Unit of Diabetology, Dietology and Clinical Nutrition, Sant’Anna Hospital, Ferrara, Italy

## Abstract

**Background:**

Cardiovascular disease (CVD) is the leading cause of death in type 2 diabetes mellitus (T2DM). American Diabetes Association standards of care set a series of targets recommended for the CVD prevention: blood pressure, LDL and HDL cholesterol (LDL-C and HDL-C), triglycerides and HbA_1c_ goals. The aim of this study was to evaluate cardiovascular risk factors in a T2DM outpatient population in order to estimate their specific clinical value in predicting long-term overall mortality.

**Methods:**

Our study population was composed of 1917 T2DM outpatients attending the hospital-based Diabetes Clinic of Ferrara for a mean follow-up period of 10 years; recorded information included personal, clinical and biochemical data, and pharmacological treatment.

**Results:**

A Cox proportional hazard analysis was performed, pointing out as age (HR:1.08; IC95%: 1.06-1.11), sex (males: HR:1.97; IC95%: 1.26-3.07), mean triglycerides levels during follow-up (III vs I tertile: HR:1.87; IC95%: 1.12-3.12) and lipid-lowering treatment (HR:0.56; IC95%: 0.35-0.90) were significantly associated with all-cause mortality, independent of confounding factors such as mean values of LDL-C, HDL-C, HbA_1c_, blood pressure, BMI, fasting glucose, and antihypertensive and antidiabetic treatment.

**Conclusions:**

This finding suggests that more attention should be given to the management of cardiovascular risk in type 2 diabetic patients with high triglycerides levels.

## Background

Cardiovascular disease (CVD) is the leading cause of morbidity and mortality in individuals affected by diabetes mellitus; moreover, the rates of CVD mortality are two to four times higher in diabetic patients compared with non diabetic population [1]. Usual risk factors for coronary artery disease (CHD) account for only 25-50% of increased atherosclerotic risk in diabetes mellitus [2]. Despite achieving targets for low-density lipoprotein cholesterol (LDL-C), blood pressure and glycemia according to current standards of care, patients with dyslipidaemia remain at high residual risk of vascular events [3]. The typical pattern of diabetic dyslipidaemia, consisting of elevated triglycerides, low high-density lipoprotein cholesterol (HDL-C), and the predominance of small dense low-density lipoprotein particles, may contribute to increased CVD mortality in diabetic subjects [4]. Moreover, in diabetes lipoprotein abnormalities often appears during the asymptomatic prodromal phase of the disease, while hyperglycemia is a late stage in the sequence of events from insulin resistance to frank diabetes.

The clinical conditions commonly associated with type 2 diabetes, such as hypertension and dyslipidaemia, are critical risk factors for CVD, and diabetes itself confers an independent risk. Many studies have drown attention to the efficacy of managing single cardiovascular risk factors in preventing or delay CVD in diabetic subjects; [5] for example, LDL-C has been firmly established as a good predictor of CHD, and statins mega-trials demonstrated that an aggressive LDL-C reduction strategy effectively reduces cardiovascular complication. Although this findings, the “residual” risk remains high [6,7].

The 2013 American Diabetes Association (ADA) standards of care set a series of goals recommended for CVD prevention in subjects affected by type 2 diabetes: 1. systolic blood pressure <130 mmHg and diastolic blood pressure <80 mmHg; 2. LDL-C <100 mg/dL; 3. triglycerides levels <150 mg/dL; 4. HDL-C >40 mg/dL in men and >50 mg/dL in women; 5. HbA_1c_ goal of <7%, with best outcomes obtained when multiple risk factors are globally addressed [8]. The more recent 2014 standards of care [9] still call for an aggressive treatment strategy to reduce LDL-C, blood pressure, and HbA_1c_ in diabetic patients. Triglycerides levels <150 mg/dL and HDL-C >40 mg/dL in men and >50 mg/dL in women remains desirable; however, combination therapy with drugs adressing trygliceride and HDL-C levels is not recommeded, since data concerning the management of high triglycerides levels and low HDL-C levels remains inconclusive [10,11], and LDL-C–targeted statin therapy is the preferred strategy. Nonetheless, combination therapy has been shown not to provide additional cardiovascular benefit above statin therapy alone, and is not generally recommended [12].

The aim of the present study was to evaluate a number cardiovascular risk factors and metabolic parameters in the type 2 diabetic outpatient population of Ferrara (Italy) in order to estimate their specific clinical value in predicting long-term overall mortality.

## Methods

We collected data from a sample of 1917 type 2 diabetic outpatients attending, for the first time, the hospital-based Diabetes Clinic of Ferrara between 1^st^ January 1996 and 31^st^ December 2006, and subsequently followed until 31^st^ May 2012.

The recorded information included the following data:

1. Personal, clinical, and lifestyle data including weight, height, body mass index (BMI), systolic and diastolic blood pressure, and smoking habit.
2. Clinical history and information relevant to microvascular (retinopathy, nephropathy and neuropathy) and macrovascular (CHD, cerebrovascular disease, and peripheral arterial disease) complications. Subjects were considered hypertensive when having mean systolic blood pressure ≥140 mmHg and/or mean diastolic blood pressure ≥90 mmHg and/or when they were on active antihypertensive treatment. Patients were defined as dyslipidaemic, according to National Cholesterol Education Program Adult Treatment Panel III 2004 Guidelines, [13] when having mean total cholesterol ≥200 mg/dl and/or mean LDL-C ≥130 mg/dl and/or mean HDL-C <40 mg/dl for males/<50 mg/dl for females, and/or mean triglycerides ≥200 mg/dl and/or when on active lipid-lowering treatment.
3. Routine biochemical data including fasting blood glucose (FBG), glycated haemoglobin (HbA_1c_), total cholesterol (TC), triglycerides (TG), HDL-C, LDL-C (by the Friedewald equation: TC – HDL-C – TG/5), and creatinine levels at baseline and during follow-up (minimum, maximum, and mean value). Mean values were calculated upon all records during follow-up, with a mean number of 10 visits for each patient (range: 6 to 15 visits).
4. Pharmacological treatment including antihypertensive, antidiabetic, and lipid lowering drugs (statins and/or fenofibrates and/or omega-3 fatty acids).

All deaths during the period of follow-up were recorded in two ways: 1. direct contact by telephone with the family of the patient; 2. direct control of the living status on provincial death register. However, data about the specific cause of death were not available for the study.

### Statistical analysis

Continuous variables were expressed as mean value ± standard deviation (SD) or as median value (interquartile range), when appropriate. Categorical variables were expressed as percentages. Differences between groups were assessed by the Student’s *t* test for continuous variables, the Kruskal-Wallis test for continuous variables without normal distribution, the *x*^*2*^ test for the categorical data. Multivariate Cox proportional hazard analysis was used to examine the impact of different metabolic parameters and treatments on the Hazard Ratio (HR) for total mortality (method: stepwise backward). The assumption of proportionality of all variables introduced in the models was assessed through the analysis of Schoenfeld residuals. The Cox models were adjusted for other factors associated with mortality. Two different models were tested:

- Model 1 included: age and gender; tertiles of BMI; mean values of fasting blood glucose, HbA_1c_, systolic blood pressure, serum LDL-C, HDL-C, and triglycerides; antihypertensive drugs (yes/no), lipid-lowering drugs (yes/no), and antidiabetic treatment. For the latter, five categories were compared with diet (reference group), including 1. biguanides, 2. sulfonylureas, 3. biguanides + sulfonylureas, 4. insulin, and 5. insulin + oral hypoglycemic agents.
- Model 2 included: age and gender; HbA_1c_, blood pressure, LDL-C, HDL-C and triglycerides according to the ADA goal categories; antihypertensive drugs, lipid-lowering drugs, and antidiabetic treatment (for categorization see Model 1). Subjects were categorized into 3 groups according to the achievement of ADA recommended target for blood pressure, HbA_1c_, HDL-C, LDL-C, and triglycerides:

1. “satisfying criteria” when mean value and all recorded values of the variable satisfied the ADA goal during the whole follow-up (reference group);
2. “partially satisfying criteria” for those individuals failing to achieve the ADA goal in one or more situations during follow-up, despite having a mean value reaching the ADA goal;
3. “not satisfying criteria”: all other subjects.

Analyses were performed by SPSS for Windows statistical package, version 13.0.

Our research is in compliance with the Helsinki Declaration. No informed consent was required because this study is observational and did not affect patient care in any way.

## Results

### Population characteristics

At baseline, the mean age of subjects was 58.0 ± 10.1 SD years; 40.6% were female. During the 10 years follow-up period, there were 95 deaths, with a total mortality rate of 5%. As expected, the deceased group (D) was older and showed a larger proportion of men (66.3%) compared with the survivors group (S). The principal characteristic of the sample at baseline, according to the outcome (alive/deceased) are summarized in Table 1. Compared to S group, subjects included in D group had lower systolic (133 vs 136 mmHg) and diastolic blood pressure (77 vs 79 mmHg), and higher creatinine levels (1.2 vs 0.9 mg/dl). The principal follow-up characteristic of the sample, according to the outcome are summarized in Table 2. Compared to S group, subjects belonging to D group were characterized by lower mean diastolic blood pressure (79 vs 80 mmHg), higher mean creatinine levels (1.1 vs 0.9), and higher prevalence of diabetic dyslipidaemia and microvascular/macrovascular complications, although only nephropathy and retinopathy were statistically significant (37 vs 17%, and 17 vs 8%, respectively). Moreover, subjects in D group were more often treated with insulin (23 vs 7%), and less frequently treated with lipid lowering drugs and biguanides (26 vs 38%, and 17 vs 33%, respectively), compared with group S. On the whole, 716 subjects were taking lipid lowering drugs (62.6%); 640 subjects were taking statins (534 statins only, 105 statins + omega-3 fatty acids, one statin plus fibrate), 50 subjects were taking fibrates (46 fibrates only, three fibrates + omega-3 fatty acids), and 27 subjects were taking omega-3 fatty acids only.

**Table 1:** **Baseline principal characteristics of 1917 outpatients with type 2 diabetes according to 10**-**years overall mortality**

	**Baseline**		**p**
	**Alive**	**Deceased**	
	**(N = 1822)**	**(N = 95)**	
**Female gender (%)**	41.0	33.7	0.09
**Age** **(years)**	57.7 ± 10.1	64.1 ± 9.1	**0.001**
**Weight** **(Kg)**	83.1 ± 16.5	83.2 ± 19.1	0.94
**BMI** **(Kg/m** ^**2**^ **)**	29.6 ± 5.2	29.0 ± 5.6	0.34
**Fasting glucose** **(mg/dl)**	144.0 ± 45.6	145.6 ± 57.2	0.74
**HbA**_**1c**_ **(%)**	7.4 ± 1.4	7.5 ± 1.6	0.61
**Systolic blood pressure** **(mmHg)**	136.2 ± 14.5	133.1 ± 15.2	**0.04**
**Diastolic blood pressure** **(mmHg)**	79.1 ± 6.9	77.0 ± 6.7	**0.004**
**Total cholesterol** **(mg/dl)**	190.4 ± 40.5	189.4 ± 40.4	0.81
**LDL cholesterol** **(mg/dl)**	108.5 ± 35.6	109.8 ± 34.0	0.72
**HDL cholesterol** **(mg/dl)**	50.9 ± 14.2	49.6 ± 15.6	0.39
**Triglycerides** **(mg/dl)** **°**	139.0 (102.0-190.0)	144.0 (105.0-187.0)	0.91
**Creatinine** **(mg/dl)°#**	0.9 (0.8-1.2)	1.2 (0.9-1.8)	**0.001**

°Median (interquartile range) # Not available in 552 patients.

**Table 2:** **Follow**-**up principal characteristics of 1917 outpatients with type 2 diabetes according to 10**-**years overall mortality**

	**Follow-** **up values**		**p**
	**Alive**	**Deceased**	
	**(N** ** = ** **1822)**	**(N** ** = ** **95)**	
**Weight** **(Kg)**	83.8 ± 16.4	84.7 ± 17.9	0.57
**BMI** **(Kg/m** ^**2**^ **)**	29.8 ± 5.1	29.5 ± 4.8	0.64
**Fasting glucose** **(mg/dl)**	143.3 ± 30.6	148.8 ± 36.9	0.08
**HbA**_**1c**_ ** (%)**	7.6 ± 1.2	7.7 ± 1.3	0.22
**Systolic blood pressure** **(mmHg)**	136.7 ± 11.9	136.1 ± 12.0	0.66
**Diastolic blood pressure** **(mmHg)**	80.4 ± 5.6	79.0 ± 5.2	**0.01**
**Total cholesterol** **(mg/dl)**	196.0 ± 36.1	197.5 ± 37.1	0.71
**LDL cholesterol** **(mg/dl)**	113.6 ± 32.2	114.8 ± 31.2	0.72
**HDL cholesterol** **(mg/dl)**	50.5 ± 12.8	50.0 ± 13.9	0.74
**Triglycerides** **(mg/dl)°**	147.0 (108.0-196.0)	149.5 (115.5-204.0)	0.52
**Creatinine** **(mg/dl)°#**	0.9 (0.8-1.1)	1.1 (0.9-1.7)	**0.001**
**Complications (%)**			
**- Hypertension**	71.0	74.7	0.25
**- Dyslipidaemia**	79.5	86.3	0.06
**- Coronary hearth disease**	5.8	7.4	0.32
**- Cerebro**-**vascular disease**	1.3	2.1	0.35
**- Peripheral arterial disease**	2.9	6.3	0.06
**- Nephropathy**	17.0	36.8	**0.001**
**- Retinopathy**	8.3	16.8	**0.007**
**- Neuropathy**	3.0	4.2	0.33
**Treatments (%)**			
**Antihypertensive**	55.8	63.2	0.09
**Lipid**-**lowering**	37.9	26.3	**0.01**
**Antidiabetic**			
**- Only diet**	10.0	14.7	0.21
**- Biguanides**	33.5	16.8	**0.001**
**- Sulfonylureas**	4.9	3.2	0.32
**- Biguanides** ** + ** **Sulfonylureas**	29.6	22.1	0.07
**- Insulin**	7.0	23.2	**0.001**
**- Insulin** ** + ** **OHA**	15.0	20.0	0.12

Mean values were calculated upon all records during follow-up, with a mean number of 10 visits for each patient (range: 6 to 15 visits). The total prevalence of complications and treatments were evaluated at the time of the end of the study.

°Median (interquartile range) # Not available in 552 patients.

### Cox analysis

In order to evaluate the possible impact of different variables on long-term total mortality, a Cox proportional hazard analysis was performed by constructing 2 different models (see statistical analysis). In Model 1, the HR for 10-years total mortality was associated with male gender (HR: 1.86; 95% CI: 1.20-2.88), age (HR: 1.09; 95% CI: 1.06-1.12), current use of lipid lowering drugs (HR: 0.56; 95% CI: 0.35-0.90), and serum triglycerides (HR: III vs I tertile 1.87; 95% CI: 1.12-3.13) (Table 3). As regards antidiabetic treatment, the use of biguanides (HR: 0.47; 95% CI: 0.22-0.96) or biguanides associated with sulfonylureas (HR: 0.42; 95% CI: 0.21-0.83) was associated with a reduction in mortality risk compared to diet treatment.

**Table 3:** **Hazard Ratios** (**HR**; **95**%** CI**) **for 10**-**years overall mortality in 1917 outpatients with type 2 diabetes mellitus according to the follow**-**up parameters** (**MODEL 1**)

**Model 1**	**B**	**SE**	**HR**	**95% CI**		**p**
				**Inferior**	**Superior**	
**Male gender**	.62	.22	1.86	1.20	2.88	**0.005**
**Age**	.08	.01	1.09	1.06	1.12	<**0.001**
**Lipid**-**lowering drugs**	-.57	.24	.56	.35	.90	**0.017**
**Triglycerides tertiles**						
**- I tertile**			1	-	-	0.054
**- II tertile**	.31	.26	1.37	.82	2.30	0.233
**- III tertile**	.63	.26	1.87	1.12	3.13	**0.016**
**Antidiabetic treatment**						
**- Diet**			1	-	-	**0.011**
**- Biguanides**	-.76	.37	.47	.22	.96	**0.040**
**- Sulfonylureas**	-.74	.64	.48	.14	1.67	0.247
**- Biguanides** ** + ** **Sulfonylureas**	-.86	.35	.42	.21	.83	**0.013**
**- Insulin**	.11	.35	1.11	.56	2.20	0.756
**- Insulin** ** + ** **OHA**	-.21	.37	.81	.39	1.66	0.568

In the Cox model: age, gender; tertiles of Body Mass Index; Fasting Blood Glucose, HbA_1c_, systolic blood pressure, serum LDL-C, HDL-C, and triglycerides mean values; antihypertensive drugs, lipid-lowering drugs, and antidiabetic treatment.

In Model 2, besides age and gender, the HR for overall mortality was associated with lipid lowering drugs (HR: 0.56; 95% CI: 0.35-0.91), and triglycerides ADA categories (HR: Partially satisfying vs Satisfying criteria 2.05; 95% CI: 1.14-3.68; HR: Not satisfying vs Satisfying criteria 1.73; 95% CI: 1.08-2.77) (Table 4). Again, the use of biguanides (HR: 0.45; 95% CI: 0.22-0.96) or biguanides associated with sulfonylureas (HR: 0.42; 95% CI: 0.21-0.84) was also associated with a reduction in mortality risk.

**Table 4:** **Hazard Ratios** (**HR**; **95**%** CI**) **for 10**-**years overall mortality in 1917 outpatients with type 2 diabetes mellitus according to the follow**-**up parameters** (**MODEL 2**)

**Model 2**	**B**	**SE**	**HR**	**95% CI**		**p**
				**Inferior**	**Superior**	
**Male gender**	.68	.23	1.97	1.27	3.08	**0.003**
**Age**	.09	.01	1.09	1.06	1.12	<**0.001**
**Lipid-** **lowering drugs**	-.57	.24	.56	.35	.91	**0.019**
**Triglycerides ADA categories**						
**- Satisfying criteria**			1			**0.025**
**- Partially satisfying criteria**	.72	.30	2.05	1.14	3.68	**0.017**
**- Not satisfying criteria**	.55	.24	1.73	1.08	2.77	**0.022**
**Antidiabetic treatment**						
**- Diet**			1			**0.006**
**- Biguanides**	-.79	.38	.45	.22	.96	**0.038**
**- Sulfonylureas**	-.73	.64	.48	.14	1.68	0.253
**- Biguanides** **+** **Sulfonylureas**	-.86	.35	.42	.21	.84	**0.014**
**- Insulin**	.17	.35	1.18	.60	2.33	0.630
**- Insulin** **+** **OHA**	-.18	.37	.84	.41	1.72	0.631

In the Cox model: age, gender; HbA_1c_, blood pressure values; serum LDL-C, HDL-C and triglycerides categorized by ADA criteria; antihypertensive drugs, lipid-lowering drugs, antidiabetic treatment.

## Discussion

In this study we evaluated the relative impact of well-established CVD risk factors in a type 2 diabetic outpatient population, and estimated their specific value in predicting long-term all-cause mortality. Indeed, not only patients with diabetes have a greater burden of atherogenic risk factors compared with non-diabetic individuals, but many of these are already present in the early stages of the disease. In particular, diabetic subjects have a peculiar pattern of dyslipidaemia characterized by elevated triglycerides, low HDL-C, and presence of small-dense LDLs. This pattern can be often detected before the onset of overt hyperglycaemia, and it is thought to be secondary to the presence of hyperinsulinemia and insulin resistance [14] Multiple risk factor interventions (reducing glucose, lipids, blood pressure, etc.) have been shown to be effective in reducing all-cause and CVD mortality in different trials, and should be enforced in all patients with type 2 diabetes [15]. As a matter of fact, current guidelines (ADA and EASD) call for an aggressive treatment strategy in order to reduce glucose levels, blood pressure, and LDL-C in diabetic patients, while triglycerides levels <150 mg/dL and HDL-C >40 mg/dL in men and >50 mg/dL in women are only considered desirable ad not therapeutic goals [16,17]. Our longitudinal data gave us the opportunity to explore the possible effect of achieving the therapeutic ADA goals and satisfying desirable triglycerides levels on all-cause mortality.

### Serum Triglycerides and 10 years mortality

The contribution of triglycerides to CVD risk has been much debated in the past, with many important prospective studies observing an association between elevated triglycerides levels and CVD risk (in particular coronary risk) [18-20], but also risk for microangiopathy [21]; nevertheless, more recent studies gave different results, suggesting that this association might be weakened when adjustment for other risk factors was made [22]. The pathogenetic mechanism undergoing these findings remains uncertain, and could be mainly related to triglycerides induced endothelial dysfunction through oxidative stress. By Cox analysis (Model 1) we demonstrated a direct association between long-term mortality risk and triglycerides levels; the association was strong and significant even after multivariate adjustment for traditional CVD risk factors including BMI, HbA_1c_, LDL-C, and medication use. This independent association with long term all-cause mortality support the idea that serum triglycerides could play a role in type 2 diabetic patients mortality risk [23].

Treating residual risk by lowering triglycerides is still debated at present time [24]; infact, it is not clear whether or not a pharmacological intervention targeted to reducing triglycerides levels would contribute to a cardiovascular risk reduction [25]. Dedicated trials failed to find a significant reduction in CVD outcomes in a diabetic population treated with fenofibrates, [26] while meta-analyses conducted upon pre-specified subgroups have confirmed the clinical benefits of fibrates on major CVD events [27,28]. A randomized clinical trail with statin therapy plus extended release niacin including over 3,000 patients (one-third with diabetes) with established cardiovascular disease, low levels of HDL cholesterol, and triglycerides levels of 150–400 mg/dL, was halted early due to lack of efficacy on the primary cardiovascular disease outcome, and a possible increase in ischemic stroke in those on combination therapy [29]. Hence, combination lipid-lowering therapy cannot be broadly recommended. However, ADA guidelines state that triglycerides concentrations below 150 mg/dL are desirable. According to this, in our population, the satisfaction of this “desirable” triglycerides level was associated with a lower 10 years total mortality. Thereby, we suggest that more attention should be given to cardiovascular risk management in type 2 diabetic patients with high triglycerides levels; specifically, a more strict management of the other modifiable risk factors (e.g. diet, physical exercise, blood pressure, LDL-C goal) could be indicated.

As a matter of fact, the second Cox analysis was modelled upon accomplishment of ADA criteria (Model 2), and confirmed the previous observation; among the criteria established for CVD prevention in patients with type 2 diabetes, only triglycerides desirable level criteria (<150 mg/dl) was significantly associated with a lower mortality risk in our population.

More interesting, diabetic patients belonging to the “partially satisfying criteria” group (reached triglycerides target for mean values, but failure in one or more follow-up visit) had a higher HR for mortality (HR: 2.02; 95% CI: 1.11-3.48) compared to the “satisfying criteria” reference group (always reached target); besides, their HR for total mortality was very similar to the “not satisfying criteria” group (HR: 1.83; 95%IC: 1.08-2.97). We are not sure about the nature of this finding in the “partially satisfying criteria” group, and there are at least two possible explanations: 1. the atherogenic process might be activated by hypertriglyceridemia, but might successively continue independently from the persistence of high fasting triglycerides; 2. in this group, unmeasured fluctuations of triglycerides levels might be present, and might be as atherogenic and dangerous as high fasting triglycerides levels, thus predisposing them to a similar increase in the risk [30].

### Lipid-lowering drugs and 10 years mortality

CHD is the most important cause of morbidity and mortality among subjects with diabetes mellitus. Statins mega-trial have demonstrated that LDL-C reduction is effective in reducing cardiovascular mortality in diabetic subjects; [31] therefore, LDL-C reduction is one of the primary therapeutic goals in reducing cardiovascular risk in diabetes [32]. According to these findings, our data show that the lipid lowering treatment is significantly associated with a reduced mortality risk, and this phenomenon is independent from other variables included into the model (HbA_1c_, blood pressure, LDL-C, HDL-C, and triglycerides values). The vast majority of subjects taking lipid lowering drugs were assuming statins (90%); thus, this class of drugs is most likely to be responsible of the reduced mortality, independent from the known effect on serum cholesterol levels. Once more, there might be evidence of the “very discussed” pleiotropic effects of statins [33]; these effects may include reduced endothelial inflammation, nitric oxide production enhancement, and improved insulin sensitivity [34]. Important clinical trials such as WOSCOPS [35] and CARE [36] indicated that, despite comparable serum cholesterol levels, patients treated with statin had lower risk of CVD events compared with the placebo groups. Most of these effects might be mediated by the inhibition of isoprenoid synthesis, which affects multiple signalling pathways.

### Biguanides and 10 years mortality

Metformin is considered the first line pharmacological therapy in type 2 diabetes [37,38]. Its main effect is enhancement of cell insulin sensitivity [39]. Important studies, such as the UK Prospective Diabetes Study (UKPDS), have demonstrated a significant effect of metformin therapy on overall mortality risk reduction, and particularly on CVD and myocardial infarction outcomes [40]. Other clinical and experimental studies have shown that metformin treatment might be associated with improved outcomes, supporting the conclusions from UKPDS. In addition, a well-designed retrospective analysis has shown significantly lower mortality rates in patients receiving metformin compared with patients treated with sulphonylurea monotherapy [41]. The ADA and EASD consensus guidelines on the management of hyperglycemia in type 2 diabetes explicitly state that metformin should be used as first-line therapy, in addition to lifestyle interventions. Our data are consistent with this indication, showing that metformin treatment is associated with a reduced mortality risk compared to diet and other antidiabetic agents. Nevertheless, we have to make some considerations upon this result. In particular, patients treated with metformin had, on average, lower creatinine levels compared with controls (0.96 ± 0.31 vs 1.12 ± 0.63). Thus, although this finding is not surprising (metformin cannot be prescribed in subjects with chronic kidney disease), the difference in renal function might contribute to the results.

Finally, we have to acknowledge some important limitations of the study. First, our laboratory data consisted of only minimum, maximum, and mean values, while the single laboratory determinations were unavailable. Second, we disposed of data concerning presence/absence of micro/macro vascular complications, but we didn’t have precise additional information upon the onset of cardiovascular events. Third, we only disposed of all-cause mortality without any information concerning the possible cause of death. We would also underline the strength of our clinical study; indeed, our results are based on a large sample of older adults type 2 diabetic outpatients (1917 individuals, 40% females) with a long-term follow-up (on average ten years).

## Conclusions

In conclusion, we found a direct association between mean triglycerides levels and long-term total mortality risk in older adult type 2 diabetic outpatients; the relationship was significant even after taking into account for the effect of traditional cardiovascular risk factors and pharmacological treatments. This finding suggests that more attention should be given to cardiovascular risk management in type 2 diabetic patients with high triglycerides levels.
